# Congenital myasthenic syndrome in a cohort of patients with ‘double’ seronegative myasthenia gravis

**DOI:** 10.1590/0004-282X-ANP-2020-0575

**Published:** 2021-12-31

**Authors:** Paulo José Lorenzoni, Renata Dal-Pra Ducci, Raquel Cristina Arndt, Nyvia Milicio Coblinski Hrysay, Otto Jesus Hernandez Fustes, Ana Töpf, Hanns Lochmüller, Lineu Cesar Werneck, Cláudia Suemi Kamoi Kay, Rosana Herminia Scola

**Affiliations:** 1 Universidade Federal do Paraná, Hospital de Clínicas, Departamento de Clínica Médica, Serviço de Doenças Neuromusculares, Curitiba PR, Brazil. Universidade Federal do Paraná Hospital de Clínicas Departamento de Clínica Médica Curitiba PR Brazil; 2 Newcastle University, Institute of Genetic Medicine, John Walton Muscular Dystrophy Research Centre, Newcastle upon Tyne, UK. Newcastle University Institute of Genetic Medicine John Walton Muscular Dystrophy Research Centre Newcastle upon Tyne UK; 3 University of Ottawa, Children's Hospital of Eastern Ontario Research Institute, Department of Medicine, Division of Neurology, Ottawa, Canada. University of Ottawa Children's Hospital of Eastern Ontario Research Institute Department of Medicine Ottawa Canada; 4 University of Ottawa, The Ottawa Hospital, Brain and Mind Research Institute, Ottawa, Canada. University of Ottawa The Ottawa Hospital Brain and Mind Research Institute Ottawa Canada

**Keywords:** Myasthenic Syndromes, Congenital, Myasthenia Gravis, Genetics, Síndromes Miastênicas Congênitas, Miastenia Gravis, Genética

## Abstract

**Background:**

Congenital myasthenic syndromes (CMS) have some phenotypic overlap with seronegative myasthenia gravis (SNMG).

**Objective:**

The aim of this single center study was to assess the minimum occurrence of CMS misdiagnosed as double SNMG in a Brazilian cohort.

**Methods:**

The genetic analysis of the most common mutations in *CHRNE*, *RAPSN*, and *DOK7* genes was used as the main screening tool.

**Results:**

We performed genetic analysis in 22 patients with a previous diagnosis of ‘double’ SNMG. In this study, one CMS patient was confirmed due to the presence of compound heterozygous variants in the *CHRNE* gene (c.130insG/p.Cys210Phe).

**Conclusions:**

This study confirmed that CMS due to *CHNRE* mutations can be mistaken for SNMG. In addition, our study estimated the prevalence of misdiagnosed CMS to be 4.5% in ‘double’ SNMG patients of our center. Based on our findings, genetic screening could be helpful in the diagnostic workup of patients with ‘double’ SNMG in whom differential diagnosis is recommended.

## RESUMO

**Antecedentes:**

As síndromes miastênicas congênitas (SMC) podem ter sobreposição fenotípica com a miastenia gravis soronegativa (MG-SN).

**Objetivo:**

Estabelecer a prevalência mínima de SMC diagnosticada inicialmente como MG duplo soronegativa em uma série de casos brasileiros.

**Métodos:**

A análise genética das mutações mais comuns nos genes *CHRNE*, *RAPSN* e *DOK7* foi usada como o principal exame de triagem.

**Resultados:**

Vinte e dois pacientes com diagnóstico prévio de MG-SN foram geneticamente analisados, sendo que uma paciente foi confirmada com SMC devido a presença de variante em heterozigose composta no gene *CHRNE* (c.130insG/p.Cys210Phe).

**Conclusões:**

O presente estudo confirma que SMC devido mutação no gene *CHNRE* pode ser inicialmente diagnosticada como MG-SN. O estudo estimou como 4,5% a prevalência de diagnóstico de SMC entre nossos pacientes préviamente diagnosticados como MG-SN. Com base nesse estudo, a análise genética pode ser recomendada para investigação do diagnóstico diferencial em pacientes com MG-SN.

## INTRODUCTION

Congenital myasthenic syndromes (CMS) are heterogeneous inherited diseases caused by specific mechanisms that compromise the function of neuromuscular transmission^1^^,^^2^. Some CMS patients present clinical manifestation from birth or shortly after, whereas others, especially those with mild presentations, go undiagnosed until adolescence or adulthood^1^^-^^5^. CMS are usually identified by clinical manifestations, family history, electrophysiologic studies, and response to acetylcholinesterase inhibitors^1^^,^^2^. The presence of an affected family member seems to be the strongest indication to initially suspect of CMS. However, in sporadic patients with no reported affected family, other signs can help the diagnosis: age at onset, delayed motor development, hypotonia, ptosis, ophthalmoplegia, weakness that may worsen on exertion, skeletal deformities (e.g. arthrogryposis, lordosis, or scoliosis), standard response to the use of acetylcholinesterase inhibitors, and no response to treatment with immunosuppressants^1^^,^^2^. 

CMS and MG share many clinical and electrophysiological features; thus, they can be difficult to differentiate, mainly when they present in adolescents or adults^3^^-^^5^. An abnormal decrement on low-frequency repetitive nerve stimulation (RNS) or an increased jitter on single-fibre electromyography (SFEMG) confirms an underlying neuromuscular transmission defect in CMS, but these electrodiagnostic findings are similar to those in MG^6^^,^^7^. This fact makes it difficult to electrophysiologically differentiate CMS from MG^6^^,^^7^. In this situation, the presence of serum antibodies, e.g. anti-acetylcholinesterase receptor (AChR), is helpful to distinguish between the diseases. However, this situation is still challenging, especially if the initial diagnosis is seronegative MG (SNMG), which is usually one cause of the delay in the CMS diagnosis in pediatric and adult populations^4^^-^^6^^,^^8^^,^^9^. 

CMS was previously misdiagnosed as SNMG^3^^-^^7^. CMS and MG are treated differently, and the recognition of such cases is important in order to ensure beneficial therapy for patients and prevent the use of inappropriate immunosuppression^3^^,^^6^^,^^10^. The main objective of this study was to assess the prevalence of CMS in a Brazilian population diagnosed as ‘double’ SNMG (absence of serum antibodies against AChR and muscle-specific tyrosine kinase [MuSK]). To investigate this, we screened a targeted panel, including hot-spot mutations previously identified in Brazilian patients, to detect the CMS.

## METHODS

We selected all cases catalogued as ‘double’ SNMG who visited a single neuromuscular disorder center at Hospital de Clínicas of the Universidade Federal do Paraná (Curitiba, Brazil) between 2015 and 2019. We included MG patients who met the clinical (including purely ocular symptoms), laboratorial (‘double’ absence of serum antibodies), and electrophysiological (compound muscle action potential with decrement greater than 10% in 3 Hz repetitive nerve stimulation in at least one site) diagnostic criteria for SNMG. We excluded patients with suspected CMS, relatives of CMS patients, relatives of MG patients, relatives of patients with known neuromuscular disorders and patients younger than 18 years. 

We performed a retrospective analysis of clinical data, treatment, laboratory and electrophysiological features. Relevant data, including age, gender and repetitive nerve stimulation findings, were recorded during the investigation of MG. We reviewed clinical data when a patient had a mutation in the *CHRNE*, *RAPSN,* or *DOK7* genes.

We performed molecular analysis (genetics) for the hot-spot mutations identified in the international literature by using blood specimens. We collected blood samples from peripheral veins in ethylenediaminetetraacetic acid (EDTA)-coated vacuum tubes. We extracted DNA from peripheral blood lymphocytes using a modified phenol/chloroform method.

We analyzed the point mutations c.130insG, c.1327delG, and c.1353insG, respectively in exon 2, 11, and 12 of the *CHRNE* gene, by Sanger sequencing. In the sequencing, we used two sets of oligonucleotides to amplify the putative DNA mutations in the exon 2 (F-5’-CAGTGAGATGAGATTCGTCAG-3’ and R-5’-CCTCACACAGGCACCCTGGCA-3’) and exons 11 to 12 (F-5’-CTGGAGATGGGTGGGAAATTG-3’ and R-5’-CACGGAGCGAGCTCGTGTTTGA-3’) by two conventional polymerase chain reactions (PCRs) with Taq DNA polymerase. The PCRs produced 518 base-pair (bp; for exon 2) and 550 bp (for exons 11 and 12) fragments that were purified and sequenced.

We analyzed the point mutation p.N88K (c.264C>A; p.Asn88Lys) in exon 2 of the *RAPSN* gene by Sanger sequencing. In the sequencing, we used oligonucleotide primers (F-5’-GCCACAGGGTGTGTGCCTCA-3’ and R-5’-AGGCTGGGGTCCAAGGCTCAGAGT-3’) to amplify the putative DNA mutation by conventional PCR with Taq DNA polymerase. The PCR produced a 476 bp fragment that was purified and sequenced. 

We analyzed the frameshift mutation c.1124_1127dupTGCC (p.Ala378SerfsTer30) in exon 7 of the *DOK7* gene by Sanger sequencing. In the sequencing, we used oligonucleotide primers (F-5’-AGCAATCCTCGTCGTCAGCCAGCAC-3’ and R-5’AAGAAAGCCGGGGGTGGCCCCGCGTG-3’) to amplify the putative DNA mutation by conventional PCR with Taq DNA polymerase. The PCR produced a 610 bp fragment that was purified and sequenced. 

We used a Big Dye Terminator Cycle Sequencing Kit (Applied Biosystems) and an ABI PRISM 3100 Avant Genetic Analyzer (Hitachi High Technologies Corporation, Tokyo, Japan) for sequencing. We compared the obtained sequences with the revised genomic reference of these genes (*CHRNE*, *RAPSN* and *DOK7*). If the patient was homozygous for one of hot-spot mutations, CMS was confirmed. If the patient was heterozygous for one of the hot-spot mutations, as these CMS subtypes have autosomal recessive inheritance, we additionally amplified and sequenced the whole targeted gene (coding sequence and flanking intronic regions; methods available under request). 

The local ethics committee (Hospital de Clínicas da UFPR) approved the study. We obtained the informed consents for DNA tests from participants in the out-patient clinic. We conducted all studies in accordance with ethical principles after obtaining patient informed consent. 

## RESULTS

We found 22 patients with ‘double’ SNMG from unrelated families in our center who were eligible for genetic screening for CMS. The sample population comprised 15 females and 7 males, aged 19 to 69 years (mean: 45.13 ± 13.24 years; median: 43 years). The age at onset varied between 4 and 60 years (mean: 27.81 ± 13.95 years; median: 27 years). The disease duration varied between 5 and 37 years (mean: 16.86 ± 7.98 years; median: 15.5 years). The clinical presentation of SNMG was ocular in two patients and generalized in 20 patients. The MG composite scores at the last appointment ranged from 0 and 21 (mean: 5.81 ± 6.41; median: 3.5). All patients received symptomatic treatment with the acetylcholinesterase inhibitor pyridostigmine. Twenty patients used immunosuppression concomitant to symptomatic treatment: prednisone in seven patients, azathioprine in two patients, and prednisone associated with azathioprine in eleven patients. Five patients previously underwent thymectomy; the thymus histopathology revealed thymoma in three patients and thymic atrophy in two patients. 

All 22 ‘double’ SNMG patients were genetically evaluated. Genetic analysis revealed no hot-spot mutation for the *RAPSN* and *DOK7* genes in any patients (Table 1). The heterozygous c.130insG variant in *CHRNE* exon 2 was detected in only one patient (Table 1). In this patient, we sequenced the entire *CHRNE* gene; we also found the c.630G>T variant (p.Cys210Phe; g.4901163C>A) in exon 7 (Table 1). For these *CHRNE* variants, it was not possible to analyze the segregations status.

**Table 1.** Synopsis of studies in seronegative myasthenia gravis (SNMG) cohorts using genetic screening for congenital myasthenic syndromes (CMS).

Year of publication, Country	Genetic screening method	Number of investigated SNMG patients (number of confirmed CMS patients)	Misdiagnosed as CMS
2011, Norway^2^	Sanger sequencing: targeted panel for *RAPSN* (p.N88K) and *DOK7* (c.1124_1127dupTGCC) genes	74 (1): homozygous for *RAPSN* gene (p.N88K)	1.4%
2016, Australia^6^	Whole exome sequencing (followed by confirmatory Sanger sequencing)	25 (7): 3 were homozygous for the RAPSN p.N88K; 2 for *RAPSN* (S201N/E162K)*; 2 for CHRNA1 (F256L/R55H)	28%
This study, Brazil	Sanger sequencing: targeted panel for *CHRNE* (c.130insG, c.1327delG and c.1353insG); *RAPSN* (p.N88K) and *DOK7* (c.1124_1127dupTGCC) genes	22 (1): compound heterozygous for *CHRNE* gene (c.130insG/p.Cys210Phe)	4.5%
Total		121 (9)	7.4%

*One patient had a sibling with a confirmed *RAPSN* mutation (S201N/E162K), but he was not genetically tested.

**Figure 1.**
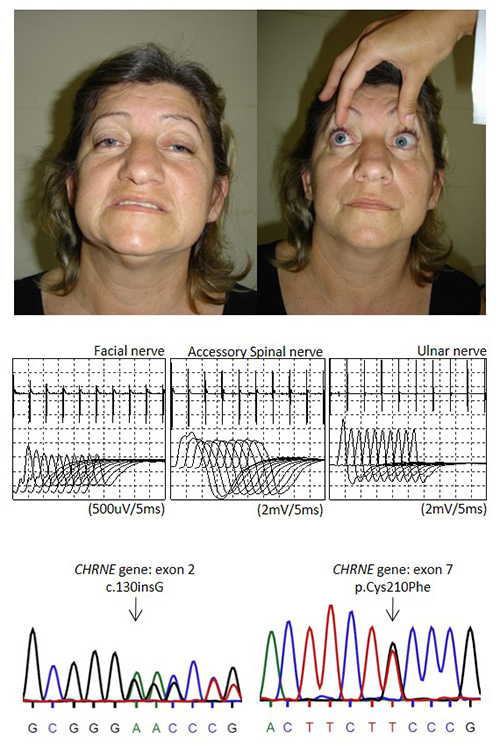
Patient with CHRNE-congenital myasthenic syndrome (CMS) showing eyelid ptosis associated with ophthalmoparesis (published with written patient consent); repetitive nerve stimulation at 3 Hz with decrement greater than 10% in the compound muscular action potential; and Sanger sequencing (electropherogram) with compound heterozygous pathogenic variants (c.130insG/p.Cys210Phe) in *CHRNE* gene.

The confirmed CHRNE-CMS patient was a 53-year-old woman who presented mild eyelids ptosis that was slowly worsening and was progressively associated with facial and proximal limb-girdle weakness since adulthood. There were no delayed motor milestones or relatives with similar symptoms. She had no osteoskeletal changes. At 33 years of age, she presented worsening of all symptoms, which were associated with dysphagia after pregnancy and a lung infection episode. At 42 years of age, the neurological examination showed eyelids ptosis, ophthalmoparesis, facial weakness, and symmetrical weakness in proximal muscles in the upper and lower limbs (Medical Research Council grade 3; Figure 1). At this time, she did not have clinical suspicion of CMS and her initial diagnosis was MG. The investigation yielded the following results: absence of serum antibodies against AChR (0.2 nmol/L; negative: < 0.45); increased serum creatine kinase (CK) levels (717 U/L; normal: < 200); normal lactic acid (1.9 nmol/L; normal: < 2); repetitive nerve stimulation with decrement response of the compound muscular action potential found in the facial, accessory spinal and ulnar nerves; and muscle biopsy with ‘ragged red fibers’ and sub-sarcolemmal accumulation of mitochondria that were compatible with mitochondrial dysfunction. Initial treatment for SNMG (pyridostigmine and prednisone) seemed to be beneficial, but her symptoms did not completely improve after some months. At 43 years of age, she presented mild deafness. Despite the immunosuppressive treatment, her disease was slowly progressing in the follow-up (MG composite: 21; QMG score: 21; MG-QOL15: 50). At that time, her clinical diagnosis was SNMG (possibly refractory to the immunosuppressive treatment) and she was undergoing genetic analysis for CMS because she met the inclusion criteria of this study (Figure 1). The investigation still showed repetitive nerve stimulation with a decremented pattern in the facial, accessory spinal, and ulnar nerves (Figure 1), serum antibodies against the AChR (0.11 nmol/L; negative: < 0.25), and MuSK (0.26 U/mL; negative: < 0.4) in the normal range. However, CHRNE-CMS diagnosis was confirmed by genetic analysis (compound heterozygous variants in the *CHRNE* gene -transcript ENST00000649488.2: c.130insG/p.Cys210Phe). 

## DISCUSSION

Clinical and electrophysiological features of CMS can easily be mistaken for MG^5^^,^^6^. Hence, it is well-established that CMS is a differential diagnosis of MG, mainly for SNMG and pediatric MG^3^^,^^8^^-^^10^. Although the worldwide prevalence of misdiagnosis is not fully known, CMS was provisionally misdiagnosed with SNMG in 9% of a pediatric cohort in England and in up to 47% of an adult cohort in North America ^6^^,^^8^. Some CMS subtypes have been highlighted as mimicking SNMG, particularly late-onset CMS due to mutations in the *RAPSN* gene (RAPSN-CMS) or limb-girdle CMS due to mutations in the *DOK7* gene (DOK7-CMS)^3^^,^^4^^,^^6^^,^^9^. Indeed, RAPSN-CMS has been reported as the most common CMS subtype that is misdiagnosed as SNMG in some cohorts^3^^,^^5^^,^^7^.

There are few studies on the prevalence of CMS that is misdiagnosed with SNMG. In SNMG investigated as potential CMS, the rate of misdiagnosed CMS was 1.4 and 28% in European and Australian cohorts, respectively (Table 1)^3^^,^^7^. Our study found a prevalence of 4.5% in adult Brazilian ‘double’ SNMG patients of our center (Table 1). In the European cohort, as in our cohort, the proportion of misdiagnosed CMS was lower than in the Australian cohort^3^^,^^7^. However, only SNMG patients with an affected sibling underwent genetic analysis in the Australian study^7^. Thus, we speculate that there may be a bias that caused the higher proportion of CMS patients with misdiagnosis in that center. In our study, we only included patients from unrelated families to avoid initially suspected CMS. In addition, as these data are usually from tertiary centers experienced in disorders that affect neuromuscular transmission, the lower proportion of misdiagnoses could be related to the high index of suspicion of CMS in the initial evaluation of the patients by these centers.

The most common form of CMS is caused by autosomal recessive mutations in the *CHRNE* gene, with hundreds of patients reported in the literature and gene-specific databases^11^. The majority of the post-synaptic CMS result from mutations within the *CHRNE* gene^11^. CHRNE-CMS mistaken for SNMG has been reported^4^. Given that CMS and MG share clinical manifestations (especially eyelids ptosis, ophthalmoparesis, and generalized weakness), our finding of a CHRNE-CMS patient mistaken for SNMG is not surprising. The c.130insG mutation, which was found as a compound heterozygous variant in our patient, is one of the most common mutations in the *CHRNE* gene for Brazilian CMS patients^12^^,^^13^. The p.Cys210Phe mutation in the *CHRNE* gene is not common; it has only been published once in association with the CMS phenotype^11^. This mutation has been described at a very low allelic frequency (ExAC Consortium: 0.000008485), predicted to be damaging by *in silico* analysis (PolyPhen-2, Mutation Tasting, UMD-Predictor) and reported to be pathogenic in gene-specific databases (LOVD, HGMD). However, *CHRNE* mutations were not found in adult patients from an Australian SNMG cohort investigated by whole exome sequencing (WES)^7^.

RAPSN-CMS has been mistaken for SNMG in European and Australian populations^3^^-^^5^^,^^7^. In a Norwegian SNMG cohort investigated for CMS, misdiagnosis occurred in 1.4%, in whom the p.N88K mutation was found to cause CMS^3^. In an Australian SNMG cohort, pathogenic variants in the *RAPSN* gene were the most frequent, and p.N88K was the commonest pathogenic variant in this gene^7^. Although none of our previous CMS cohorts of southern Brazilian patients had RAPSN-CMS, CMS cases due to a p.N88K mutation were recently reported in Brazilian patients^12^^,^^14^. Hence, we also screened p.N88K in our SNMG cohort. However, our study suggests that RAPSN-CMS mistaken for SNMG is not common in southern Brazilian patients. 

The c.1124_1127dupTGCC mutation is the most common pathogenic variant worldwide in the *DOK7* gene^15^^,^^16^. This mutation was also previously reported in Brazilian CMS patients^12^. However, we found no SNMG patients with the c.1124_1127dupTGCC mutation in our study, a result that is similar to the Norwegian and Australian cohorts^3^^,^^7^. The clinical manifestation of DOK7-CMS can appear more like a myopathy^15^^,^^16^. Therefore, DOK7-CMS has been described more as a misdiagnosis of myopathies rather than a SNMG misdiagnosis^17^^,^^18^. This factor could be one of the reasons why DOK7-CMS was not found in the published SNMG cohorts screened to CMS. 

Muscle histology of CMS patients often shows only non-specific myopathic changes^18^^-^^20^. Our patient had mitochondrial dysfunction in her muscle biopsy, which has not been reported in patients with CHRNE-CMS. Indeed, there are other CMS subtypes that are often associated with mitochondrial abnormalities in muscle biopsy (e.g. *SLC25A1*, *GFPT1* or *ALG2* genes) ^21^^-^^23^.

The advent of next generation sequencing (NGS) for the genetic diagnosis of CMS is reducing the use of Sanger sequencing as the main diagnostic tool. However, Sanger sequencing is still being performed in the investigation of CMS mostly when hot-spot mutations are previously detected in a specific population, when familial segregation is mandatory, or when NGS analysis is not available. Our study still used Sanger sequencing, which proved to be a cost-effective strategy for initial screening of our patients, as a targeted panel including the hot-spot mutations previously identified in Brazil^12^^-^^14^. Sanger sequencing as screening strategy in our study was limited to the most common mutations, i.e., the limitation of our study would be that patients with mutations that are not as common may not have been diagnosed. 

In our study, the prevalence of adult CMS was 4.5% in patients with an initial diagnosis of ‘double’ SNMG. This finding is consistent with smaller published studies, with a combined prevalence of 7.4% (Table 1)^3^^,^^7^. These differences may reflect country-specific variations in the frequency of a rare disease or bias in the selection of the SNMG patients who underwent genetic screening. Based on our findings, genetic screening by Sanger sequencing could still be helpful in the diagnostic workup of patients with ‘double’ SNMG in whom differential diagnosis is recommended. 
